# CTS trials network: Rate control vs rhythm control for atrial fibrillation after cardiac surgery - Do bitter pills have blessed effects?

**DOI:** 10.21542/gcsp.2016.15

**Published:** 2016-06-30

**Authors:** Ahmed Afifi

**Affiliations:** Aswan Heart Center, Aswan, Egypt

## Abstract

New onset AF is a very common sequel of cardiac surgery with an incidence reaching 50% in some studies. This post-operative complication leads to increased morbidity, hospital stay and, consequently, hospital costs^1^. Currently there is a great variability in the management of this condition. Despite efforts to produce best practice guidelines^2^, what best to do for a patient who develops AF post-operatively remains a question. In a systematic attempt to find an answer to this question, the Cardiothoracic Trials Network have recently published the results of their trial “Rate Control Versus Rhythm Control for Atrial Fibrillation After Cardiac Surgery”^3^ (clinicaltrials.gov number: NCT02132767).

## Introduction

The trial was conducted in 23 centers in North America. Over a period of one year 2,109 patients with similar base-line characteristics undergoing CABG and/or valve surgery without prior AF were enrolled. 695 (33%) of them had postoperative new onset AF (lasting for more than 60 minutes within one week of the operation), and of these, 523 were randomized to receive either rate or rhythm control. The primary end-point was the total number of days in the hospital 60 days after the operation. Secondary end-points included the total duration of the index hospital stay, the duration of hospitalization from AF perspective, the need for readmission, the need for permanent placement of a pacemaker, and the rates of death and adverse events.

**Figure 1.**
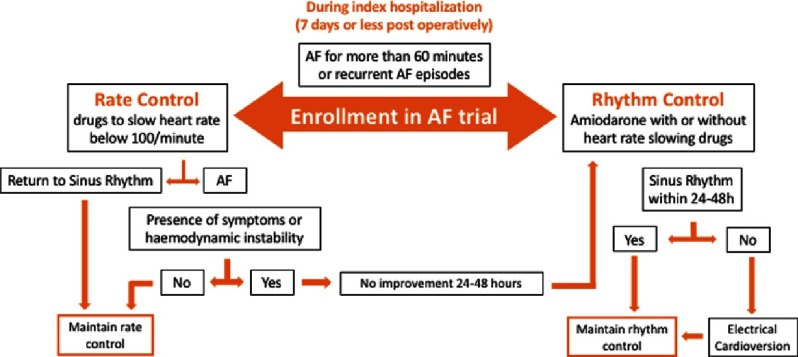
Schematic representation of the clinical patient pathway during index hospitalization^3^.

Patients in the rate-control group received medications aiming at a resting heart rate of less than 100 beats/minute. Patients in whom sinus rhythm did not return after an initial strategy of rate control were switched to rhythm control only if such treatment was found necessary to alleviate symptoms or improve hemodynamics. Patients in the rhythm-control group were treated mainly with amiodarone in addition to a rate-slowing agent when found necessary and electrical cardioversion was performed if atrial fibrillation persisted for 24 to 48 hours (Figure 1). During the course of treatment there was a 25% crossover between groups either due to drug ineffectiveness or side effects.

The primary outcome, number of days in the hospital, 60 days after randomization, did not differ between groups (median, 5.0 days for rhythm control and 5.1 days with rate control; P = 0.76) neither did the number of hospital readmissions. There was no significant difference in the rates of death, thromboembolic and bleeding events or overall serious adverse events. At hospital discharge 89.9% of the patients in the rate-control group and 93.5% in the rhythm-control group were without atrial fibrillation. At 60 days, 84.2% and 86.9% in the rate-control group and rhythm-control group, respectively, were free from AF (P = 0.41).

## Discussion

The overall incidence of post-operative AF exceeded 30% (28% after CABG only, 33.7% following valve surgery and 47.3% in combined CABG and valve surgery). At 60 days, however, 94% of the rate-control group and 98% of the rhythm control group were in steady sinus rhythm for a month (P=0.02) and respectively 84% and 87% had been free from AF since discharge. This shows that new onset AF is not only a common post-operative finding, but also a self-limiting disease that has tendency to resolve whichever way it is initially treated.

The authors, rightly, have looked at AF treatment from a point of view which matters the most in this situation; by the time AF resolves which treatment modality is associated with better resource utilization and patient experience (by measuring days in the hospital, readmissions and adverse events related to medication use or lack thereof). Although clinical end-points like stroke and bleeding would have made more impact, the number of patients required for randomization would have been prohibitive.

The rate of rehospitalization was 28% in 30 days, a fifth of which was directly due to AF recurrence. It is not very clear how AF recurrence was confirmed but it seems that only AF recurrence requiring hospitalization was accounted for. A facility for home rhythm monitoring could have provided more insight on the rate of AF recurrence. This study was carried out in post-cardiac surgery patients, a statistically similar group with a lot of clinical variability, such as type of surgery, ventricular size and function, post-operative bleeding, pericardial effusion with presence of clot and lung function. This is somewhat balanced by the large number of cases enrolled, more than ten times the previous studies^4^.

The results of this trial have come to agree with the AFFIRM study; which showed that, in non-surgical AF, rhythm control offered no survival advantage over rate control, and that rhythm control patients suffered more complications and required more hospitalization^5^, the conclusions of which lead to the strong recommendations of the joint guidelines by the American College of Cardiology, American Heart Association and Heart Rhythm Society to treat hemodynamically stable AF with beta-blockers as a first line therapy^2^. These, however were not specific to patients in the cardiac surgery setting were the evidence is lacking^6^. As with AFFIRM there was a cross over in treatment strategies in around one quarter of the patients due to hemodynamic instability or drug toxicity. There was also a high rate of discontinuation of amiodarone in the post-operative study. This highlights that treatment modality is not always optional and many times it is imposed upon the clinician by the patient condition.

## What have we learned?

The CTS Network investigators have shown us that AF is a very common occurrence after cardiac surgery but that it is also somewhat self limiting. From this study and previous studies of AF it is clear that rhythm control came at a price of drug- and cardioversion-related side effects without any overpowering clinical advantage. Rate control avoided many of the side effects of rhythm control drugs with slower conversion to sinus rhythm which didn’t seem to make a significant difference in postoperative outcomes.

This raises a number of questions; Are there other factors, like age, bleeding risk, comorbidities, diastolic dysfunction or left ventricular ejection fraction, that can sway the clinical decision? Is patient preference to be taken as a factor in the clinical decision making? Are there drugs that can offer protection against post-operative AF with minimum side effects, like beta-blockers or even quinoline chloroquine^7^? Until questions like these can be answered in a systematic fashion, balancing the trade-off between rate and rhythm control with patient factors and preference can help clinicians in treading the fine line towards improved clinical decision making and resource utilization.
